# Molecular Epidemiology of *Staphylococcus aureus* and MRSA in Bedridden Patients and Residents of Long-Term Care Facilities

**DOI:** 10.3390/antibiotics11111526

**Published:** 2022-11-01

**Authors:** Lucas Porangaba Silva, Carlos Magno Castelo Branco Fortaleza, Nathalia Bibiana Teixeira, Luís Thadeo Poianas Silva, Carolina Destro de Angelis, Maria de Lourdes Ribeiro de Souza da Cunha

**Affiliations:** 1 Department of Chemical and Biological Sciences, Biosciences Institute, UNESP—Universidade Estadual Paulista, Botucatu 18618-691, SP, Brazil; 2 Department of Infectology, Dermatology, Diagnostic Imaging and Radiotherapy, Botucatu School of Medicine, UNESP—Universidade Estadual Paulista, Botucatu 18618-970, SP, Brazil

**Keywords:** elderly population, long-term care facility, bedridden patients, *Staphylococcus aureus*, MRSA, molecular epidemiology

## Abstract

At present, multidrug-resistant microorganisms are already responsible for community-acquired infections. Methicillin-resistant *Staphylococcus aureus* (MRSA) poses a serious public health risk worldwide because of the rapid spread and diversification of pandemic clones that are characterized by increasing virulence and antimicrobial resistance. The aim of this study was to identify the prevalence and factors associated with nasal, oral and rectal carriage of *S. aureus* and MRSA in bedridden patients and residents of long-term care facilities for the elderly (LTCFs) in Botucatu, SP, Brazil. Nasal, oral and rectal swab isolates obtained from 226 LTCF residents or home-bedridden patients between 2017 and 2018 were submitted to susceptibility testing, detection of the *mecA* gene, SCC*mec* characterization, and molecular typing by pulsed-field gel electrophoresis (PFGE) and multilocus sequence typing (MLST). Logistic regression analysis was used to identify risk factors associated with the presence of *S. aureus* and MRSA. The prevalence of *S. aureus* and MRSA was 33.6% (*n* = 76) and 8% (*n* = 18), respectively. At the nine LTCFs studied, the prevalence of *S. aureus* ranged from 16.6% to 85.7% and that of MRSA from 13.3% to 25%. Living in an LTCF, male gender, a history of surgeries, and a high Charlson Comorbidity Index score were risk factors associated with *S. aureus* carriage, while MRSA carriage was positively associated with male gender. This study showed a high prevalence of *S. aureus* among elderly residents of small (<15 residents) and medium-sized (15–49 residents) LTCFs and a higher prevalence of MRSA in the oropharynx.

## 1. Introduction

The epidemiology of *Staphylococcus aureus* has undergone a conceptual revolution over recent decades [1]. This phenomenon was partly due to important changes in the epidemiological behavior of this microorganism [2]. However, old concepts were also reassessed in view of new knowledge arising from clinical and experimental research [3]. Within this context, methicillin-resistant *S. aureus* (MRSA), which have traditionally been addressed as exclusive agents of healthcare-associated [HA] infections, are now being recognized as the causative agent of severe community-acquired disease (community-associated [CA]-MRSA) [4,5].

It is estimated that about 20 to 40% of the world population are asymptomatic nasal *S. aureus* carriers and are, therefore, at increased risk of infection [6]. Colonization is a precursor stage of invasive disease. It is believed that *S. aureus* carriers are more susceptible to acquiring infection and that they are an important source of bacterial dissemination among individuals [7]. Studies have shown that colonized individuals with a high bacterial load have a six-fold higher risk of developing staphylococcal infections than non-carriers or individuals with a low bacterial load [8]. This phenomenon appears to be even more common among carriers of MRSA [9,10].

Furthermore, an important facet of the epidemiology of *S. aureus* is the involvement of “special populations”. This term refers to population strata differentiated by ecological pressures and/or specific morbidity conditions, such as elderly people and bedridden individuals. The emergence of MRSA strains poses a special risk to known vulnerable populations. Within this context, two distinct situations are of special interest: institutionalized elderly people living in nursing homes, which represent a link between the community and the hospital, and dependent (bedridden) individuals who are cared for at home and who are intermittently exposed to health services.

Elderly patients have a high morbidity and mortality risk from infectious diseases due to the presence of comorbidities, physical and cognitive disabilities, and declining immunity [11]. The risk is even higher among elderly people who are colonized with *S. aureus*, which can cause severe infection in the presence of associated comorbidities like congestive heart failure, diabetes, lung disease, and kidney failure [12]. These comorbidities are commonly found in bedridden patients cared for at home or residents of long-term care facilities for the elderly (LTCFs). One environment that can promote the acquisition and dissemination of MRSA is precisely the nursing home, where cross-transmission occurs due to permanent living in a confined environment and reduced adherence to hygienic measures as a result of cognitive impairment [13]. This fact puts residents at constant risk of colonization and infection with this microorganism. In addition, the resistance of MRSA to first-line antibiotics such as penicillin poses a risk to immunocompromised patients and makes it more difficult to treat infections [14].

Nursing homes generally admit hospitalized individuals and may therefore become a reservoir of multidrug-resistant microorganisms [15], facilitating the spread from facility to facility and leaving hospitals with low MRSA levels at risk of an outbreak if they do not maintain effective infection control [16]. Although advances in antibiotic therapy have decreased mortality, the prognosis of elderly patients infected with multidrug-resistant bacteria remains the same. Insights into the risk factors associated with the carriage and dissemination of MRSA strains in different groups of elderly people (bedridden and institutionalized) can have significant implications for the treatment and prevention of these infections.

We currently have adequate tools for studying the epidemiology of *S. aureus*, including staphylococcal cassette chromosome (SCC*mec*) typing to identify types that are more prevalent in hospital (SCC*mec* types I, II, and III) or community settings (SCC*mec* types IV and V) [4] and pulsed-field gel electrophoresis (PFGE) that allows the study of local outbreaks and can be complemented by multilocus sequence typing (MLST) for further comparisons with sequences described in the world and available in databases. The results obtained here can be used for the implementation of interventions that would permit us to reduce the spread of resistant *S. aureus* isolates among elderly people, reducing the risk of infections and complications caused by the pathogen. Furthermore, the results may help support clinical decisions aimed at improving the quality of life of this at-risk population.

We found conditions such as living in an LTCF, male gender, a history of surgeries, and a high Charlson Comorbidity Index (CCI) score to be risk factors associated with *S. aureus* carriage, while MRSA carriage was positively associated with male gender. Small- and medium-sized LTCFs were found to play an important role, with the observation of a high prevalence. Furthermore, the prevalence of MRSA was higher in the oropharynx, a site that is usually neglected. We also report for the first time the identification of ST398 in bedridden individuals and LTCF residents in Brazil.

## 2. Results

### 2.1. Prevalence of Colonization with S. aureus and MRSA

**Figure 1:**
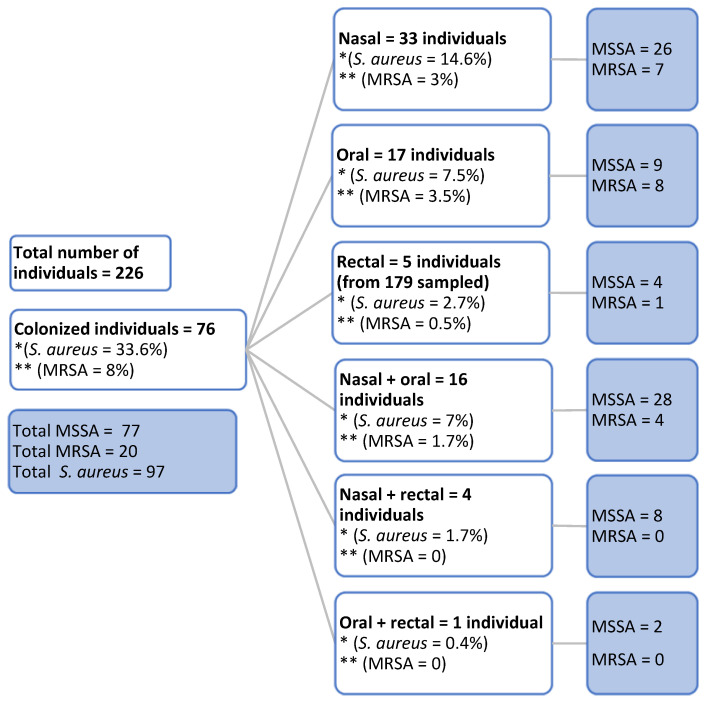
Flow chart of the total number of individuals colonized with *S.* *aureus* and number of individuals colonized with methicillin-susceptible *S. aureus* (MSSA) and methicillin-resistant *S. aureus* (MRSA) according to the sampling site. Note: Total number of individuals included in the study (226), number of individuals colonized with *S. aureus* (76), and number of colonized individuals according to the sampling site. * Overall prevalence of *S. aureus*. ** Overall prevalence of MRSA.

Samples collected from 226 individuals were analyzed. The overall prevalence of *S. aureus* carriers was 33.6% (*n* = 76), and that of MRSA carriers was 8% (*n* = 18), identified in at least one sampling site. Regarding the prevalence per site, nasal *S. aureus* carriage was identified in 53 individuals, with exclusive nasal carriage in 33 (14.6%). *S.* *aureus* was identified in oral samples of 34 individuals, with 17 (7.5%) being exclusive oral carriers. Rectal *S. aureus* was identified in 10 individuals, with exclusive rectal carriage in five (2.2%). In addition, simultaneous nasal and oral carriage was found in 16 (7%) individuals, simultaneous nasal and rectal carriage in four (1.7%), and simultaneous oral and rectal carriage in one (0.4%). Among the 20 MRSA identified, seven originated from nasal samples, eight from oropharyngeal samples, one from a rectal sample, and four simultaneously from nasal and oral samples (Figure 1). It is worth mentioning that nasal methicillin-susceptible *S. aureus* (MSSA) and oral MRSA were identified in one individual, and nasal MRSA and rectal MSSA in another.

Among the 226 individuals, 150 were from nine different LTCFs. These facilities vary in size, arbitrarily classified as small (<15 residents), medium-sized (15–49 residents) or large (50 or more residents). Table 1 shows the number of residents per LTCF, which were designated A to I.

The prevalence of *S. aureus* in each LTCF was 85.7% (six of the seven in A), 50.0% (three of the six in B), 58.3% (seven of the 12 in C), 33.3% (two of the six in D), 42.9% (three of the seven in E), 27.7% (five of the 18 in F), 33.3% (20 of the 60 in G), 50.0% (eight of the 16 in H), and 16.6% (three of the 18 in I). Residents in only four of the nine facilities were colonized with MRSA, with a prevalence of 14.3% (A), 16.7% (B), 13.3% (G), and 25% (H). Some small facilities had a higher prevalence of *S. aureus* than the other LTCFs.

Twenty (26.3%) of the 76 bedridden patients from whom samples were collected were colonized with *S. aureus*. Of these, four (5.2%) were colonized with MRSA and were mainly from the same neighborhood.

### 2.2. In Vitro Antimicrobial Susceptibility Testing

Analysis of the 97 *S. aureus* isolates from bedridden or institutionalized individuals showed that 11 isolates were resistant to oxacillin and cefoxitin, five were resistant only to oxacillin, and two were resistant only to cefoxitin, corresponding to 18 isolates with phenotypic resistance.

It is important to note that there were isolates that were resistant to oxacillin in vitro and that did not carry the *mecA* gene. In addition, some isolates were phenotypically susceptible to oxacillin and cefoxitin and carried the *mecA* gene. In this study, we classified isolates carrying the *mecA* gene as MRSA, regardless of the phenotypic result observed in the susceptibility tests.

We found no cases of resistance to sulfamethoxazole/trimethoprim, quinupristin/dalfopristin or linezolid, and the vancomycin MIC showed that all isolates were susceptible (Table 2).

### 2.3. Detection of the mecA Gene and SCCmec Characterization

The *mecA* gene was identified in *S. aureus* isolated from 18 individuals but totaling 20 samples since two individuals carried more than one resistant isolate. Thus, among the 20 resistant isolates, seven originated from nasal samples, eight from the oropharynx, one from a rectal sample, and four from both nasal and oral samples.

Among the 20 *mecA* gene-positive isolates, six carried SCC*mec* type IV (CA-MRSA), nine carried SCC*mec* type II (HA-MRSA), and one carried SCC*mec* type I (HA-MRSA). The remaining isolates could not be typed by the method of Milheiriço et al. [17].

It is important to highlight that all six SCC*mec* type IV found in the study originated from LTCF residents. The nine SCC*mec* type II were identified in three bedridden patients and in six LTCF residents. The only SCC*mec* type I was found in a bedridden patient.

### 2.4. Risk Factors for MRSA Carriage

The results of univariate and multivariate analysis (logistic regression model) for identifying risk factors associated with *S. aureus* and MRSA carriage are shown in Table 3 and Table 4.

For *S. aureus* carriage, a positive association with the time at risk (months of bedriddenness or institutionalization) was only found in univariate analysis (*p* = 0.003). In multivariate analysis, there were positive associations with living in LTCFs (OR = 2.05, 95 %CI = 1.07–3.91, *p* = 0.03), a history of surgeries in the last year (OR = 5.99, 95 %CI = 1.26–28.92, *p* = 0.02) and presence of comorbidities according to the Charlson Comorbidity Index (CCI) score (OR = 1.35, 95 %CI = 1.01–1.92, *p* = 0.047), and a negative association with heart disease (OR = 0.18, 95 %CI = 0.05–0.70, *p* = 0.01). Male gender was positively associated with *S. aureus* carriage in univariate (OR = 2.15, 95 %CI = 1.22–3.79, *p* = 0.008) and multivariate analysis (OR = 2.59, 95 %CI = 1.41–4.76, *p* = 0.002).

Regarding MRSA carriage, univariate (OR = 2.96, 95 %CI = 1.103–7.98, *p* = 0.03) and multivariate (OR = 3.29, 95 %CI = 1.18–9.17, *p* = 0.02) analysis revealed a positive association with male gender.

### 2.5. Determination of the Clonal Profile of MRSA Isolates by Pulsed-Field Gel Electrophoresis

**Figure 2:**
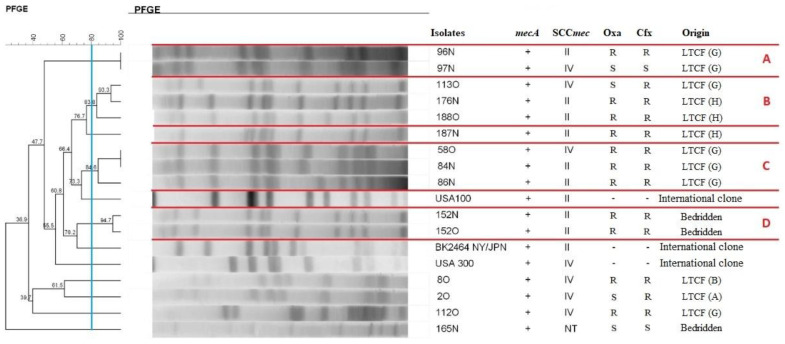
Dendrogram of PFGE-*SmaI* profiles of MRSA isolated from bedridden patients at home or long-term care facility residents generated by Dice analysis/UPGMA (BioNumerics, Applied Maths). Note: N: nasal mucosa. O: oropharyngeal mucosa. NT: not typed. Oxa: oxacillin. Cfx: cefoxitin. S: susceptible. R: resistant. LTCF: long-term care facility. ABCD letters represent clusters, lineages that showed 80% or more similarity.

**Figure 3:**
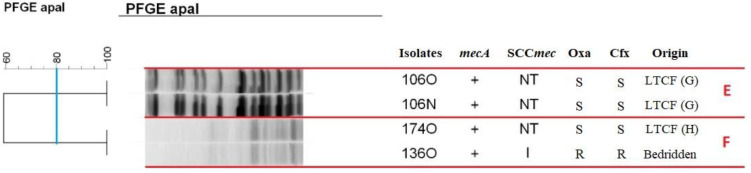
Dendrogram of PFGE-ApaI profiles of MRSA isolated from bedridden patients at home or long-term care facility residents generated by Dice analysis/UPGMA (BioNumerics, Applied Maths). Note: N: nasal mucosa. O: oropharyngeal mucosa. NT: not typed. Oxa: oxacillin. Cfx: cefoxitin. S: susceptible. R: resistant. LTCF: long-term care facility. E and F letter represent clusters, lineages that showed 80% or more similarity.

The 20 *mecA* gene-positive isolates were analyzed by PFGE. Analysis of the dendrogram revealed the presence of clusters with similarity ≥ 80% (Figure 2 and Figure 3).

Figure 2 shows the presence of four MRSA clusters after digestion with *SmaI*. Cluster A comprised two isolates from different individuals (96N and 97N) but from the same LTCF. Cluster B consisted of three isolates from different individuals (113O, 176N, and 188O), one of them living in a different LTCF. Cluster C also comprised three isolates from different individuals (58O, 84N, and 86N), all of them from the same LTCF. Cluster D consisted of two isolates (152N and 152O) from the same bedridden patient.

MRSA that was not digested with *SmaI* were submitted to PFGE typing using *ApaI* (Figure 3). Analysis of the dendrogram revealed the formation of two clusters typed with *ApaI*. Cluster E comprised two isolates (106N and 106O) from different sites of the same institutionalized individuals, while cluster F consisted of MRSA isolates from two individuals (174O and 136O), one isolated from an LTCF resident and the other from a bedridden patient.

It is important to mention that four of the five clusters identified in this study were found in facility G.

### 2.6. Molecular Typing of MRSA by Multilocus Sequence Typing

**Figure 4:**
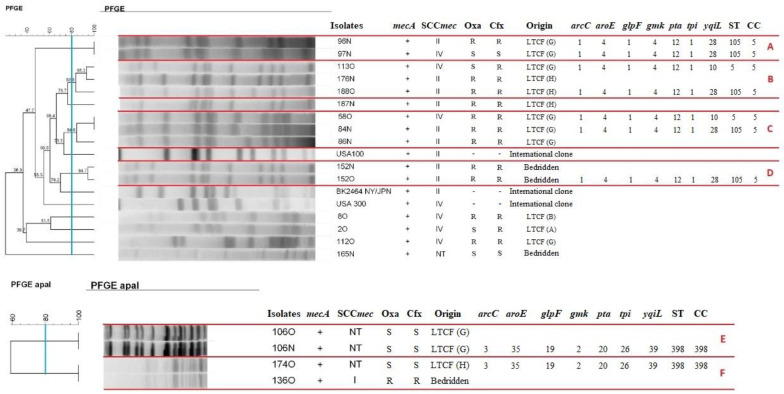
Dendrogram of PFGE-*SmaI* and PFGE-*ApaI* profiles of MRSA isolated from bedridden patients or institutionalized individuals generated by Dice analysis/UPGMA (BioNumerics, Applied Maths) and sequence types (ST) obtained by MLST. Note: Resistant isolates forming clusters >80% similarity after digestion with *SmaI* (clusters A, B, C, D) and *ApaI* (clusters E and F). N: nasal mucosa. O: oropharyngeal mucosa. LTCF: long-term care facility. *arcC*: carbamate kinase. *aroE*: shikimate dehydrogenase. *glpF*: glycerol kinase. *gmk*: guanylate kinase. *pta*: phosphate acetyltransferase. *tpi*: triosephosphate isomerase. *yqiL*: acetyl coenzyme A acetyltransferase. ST: sequence type. CC: clonal complex.

Based on the diversity found in PFGE and ensuring the choice of representative strains from each cluster identified, nine of the MRSA were selected for MLST typing. Five isolates were ST105, two isolates were ST5, and two were ST398 (Figure 4).

## 3. Discussion

A multicenter study investigating elderly nursing home residents in France found a prevalence of *S. aureus* and MRSA of 27.6% and 8.7%, respectively [18]. Another multicenter study conducted in Germany identified *S. aureus* as the most prevalent pathogenic agent among nursing home residents, with a prevalence of *S. aureus* of 29.5% and of MRSA of 1.1% [19].

Studying bedridden patients at home and LTCF residents, we found a prevalence of *S. aureus* of 33.6% and of MRSA of 8%. These percentages are similar to those reported in France [18] and Germany [19]. However, the prevalence we found here is higher compared to national data [20]. In a study conducted in a neighboring municipality 94 km away, Silveira et al. [20] observed colonization rates with *S. aureus* and MRSA of 17.7% and 3.7%, respectively, among elderly persons living in nursing homes. A similar prevalence of colonization with *S. aureus* has been reported in the population-based study by Pires et al. [21] among individuals of different ages from the same municipality (prevalence of *S. aureus* of 32.7%); however, our prevalence of MRSA (8%) was higher than that reported by these authors (0.9%).

The prevalence of *S. aureus* and MRSA in nursing homes can vary according to the number of residents. In the present study, the prevalence of MRSA was 13.3% in LTCF G (60 residents) but 25.0% in LTCF H (16 residents). Silveira et al. [20] also observed a higher prevalence of *S. aureus* and MRSA in small- and medium-sized facilities.

Among the bedridden patients at home, 20 (26.3%) of the 76 individuals were colonized with *S. aureus*. Of these, four (5.2%) were colonized with MRSA and were mainly from the same neighborhood, indicating dissemination due to proximity between houses.

Although the nasopharynx is the most consistent site of colonization with *S. aureus* and is indicated as the most appropriate site for swab screening [7], other sites (extranasal) can also be colonized. Recent studies have shown that a substantial number of individuals (7% to 32%) are oropharynx-only carriers of *S. aureus*. These findings suggest that the inclusion of a throat swab in addition to a nasal swab may be important for the success of surveillance programs [22]. Furthermore, eight of the 20 MRSA isolates of the study were only isolated from oral samples and one only from a rectal sample, highlighting the importance of extranasal sites in the epidemiology of this microorganism. These sites are commonly neglected and may contribute to the spread of the pathogen [23]. Almost half (45%) of the MRSA isolates would have been lost if other sites had not been sampled. This fact was also observed by Srinivasan et al. [24], who reported a rate of 28% of MRSA that would be lost if extranasal sites had not been sampled. The presence of MRSA at these sites also indicates a high burden and the risk of dissemination of the pathogen, with the risk of infection being significantly higher in the case of simultaneous colonization of different sites [24].

All SCC*mec* type IV strains were isolated from institutionalized individuals and were considered to be originally community-acquired [4]. SCC*mec* type II, the most prevalent in the present study, was isolated from LTCF residents and from bedridden patients, while type I was found only in one bedridden individual. These findings agree with the study by Silveira et al. [20] that found a higher prevalence of SCC*mec* type II. The authors attributed this fact to the history of hospitalizations since both type II and type I are typical of hospital-associated strains.

It is worth mentioning that individuals who are bedridden at home receive sporadic visits from health agents because of their difficulty in locomotion, a fact that can facilitate the transmission of these microorganisms. This population thus represents a link between the two environments (community and hospital) that directly influences the epidemiological dynamics of *S. aureus* and MRSA [16].

Logistic regression analysis of risk factors for colonization with *S. aureus* revealed a positive association with institutionalization. Elderly institutionalized persons live in a confined environment and often share objects with several residents, a fact that facilitates cross-transmission in these places. In addition, the frequent cognitive impairment of these individuals reduces adherence to basic hygiene measures [13]. A positive association was also observed between *S. aureus* carriage and higher median CCI scores, in agreement with the literature since the presence of a larger number of comorbidities increases the risk of colonization with *S. aureus* [12].

A history of surgeries was also positively associated with *S. aureus* carriage. Surgical site infections can be caused by microorganisms that enter the operative wound either during or after surgery, and *S. aureus* has been described as the most common cause [25]. However, this is not true when we look at the negative association observed with heart disease, which might be explained by competition with other species since 33% to 62.5% of wound infections after heart surgery are caused by coagulase-negative staphylococci [26].

Male gender was positively associated with *S. aureus* carriage and was also the only variable that showed a positive association with MRSA carriage. Studies had previously reported a higher risk of MRSA transmission among male residents of LTCFs when compared to women, probably because the former have more risk factors. In this regard, more frequent damage to the skin barrier may represent a confounder for the risk factor ‘male gender.‘ Another explanation would be biased selection in which the analyses included more men instead of case-matched controls [27,28]. Colonization may even be related to hormonal differences [29].

PFGE typing of MRSA isolated from bedridden or institutionalized individuals revealed the formation of six clusters, four of them comprising MRSA isolates from LTCF G, which had the largest number of residents as well as the largest number of MRSA isolates (9 [45%] of the 20 MRSA found in the study). Studies have shown a higher risk of colonization with MRSA across the nursing team [30], and LTCF G had a larger group of professionals than the other institutions in the study. The PFGE results also showed the formation of a cluster consisting of MRSA from LTCF G and MRSA from another medium-sized facility, indicating the spread of MRSA between different facilities. Although we cannot confirm the role of health assistants and their influence on the dissemination between the facilities since we did not investigate this issue in our study, it is important to note that different institutions tend to be attended by the same health units with a visit from the nursing team that can act as vectors. MRSA typing also revealed a similarity between one MRSA strain isolated from a bedridden patient and MRSA isolated from the medium-sized LTCF. The dissemination of persistent clones in the community requires attention since it puts the population at increased risk of infection. The different reservoirs in the community facilitate the transmission of endemic strains to households [31]. Silveira et al. [20] also found evidence of transmission between LTCFs for three clusters identified in their study.

Strain ST398 is a new livestock-associated MRSA clonal lineage that can infect or colonize humans even in the absence of exposure to livestock animals [32]. A Dutch study found that ST398 was able to spread in a nursing home, affecting seven residents and four employees [33]. Here we identified ST398 in a bedridden individual and in two residents of different LTCFs (G and H). Our study is the first to report the identification of ST398 in bedridden or institutionalized individuals in Brazil.

The data obtained may contribute to what is already known about the epidemiology of *S. aureus* and MRSA and to the identification of risk factors associated with the carriage of these microorganisms in the population studied. In conclusion, we believe that interventions are necessary in nursing homes in order to improve the quality of life and to help control the spread of these pathogens within the community. We also strongly encourage the inclusion of extranasal sites in MRSA screening.

## 4. Materials and Methods

### 4.1. Study Design

This cross-sectional study was conducted in Botucatu, SP, Brazil, where 188 LTCF residents and 222 bedridden patients were registered with the Health Department of the city. The sample size was calculated using the formula described in Appendix A, which suggested an n of 173 individuals.

The individuals were contacted during home visits and visits to the LTCF and were invited to participate in the study. The elderly persons agreed to participate by signing the free informed consent form. In the case of cognitive deficit, consent was obtained from the legal representative or, in his/her absence, from the nursing home. A questionnaire (Appendix B) containing the following data was also applied: demographic data (gender and age), time of institutionalization, clinical data (comorbidities), use of invasive devices, recent hospitalizations (last 6 months), current or recent infectious diseases, and use of antimicrobial agents (last 6 months). These data were obtained through interviews with the participants and/or their legal representatives.

### 4.2. Inclusion and Exclusion Criteria

All individuals who agreed to participate in the study were included. In the case of inability to understand, the individual was included after consent was obtained from the legal representative. Individuals institutionalized or bedridden for less than 30 days were excluded.

### 4.3. Sample Collection

Samples were collected from the nasal vestibule, oropharynx and rectum of 226 individuals (150 residents of nine LTCFs and 76 bedridden individuals at home) in the city of Botucatu, SP, Brazil, with a sterile swab specific for each site. Because of the refusal of 47 individuals, only 179 rectal swabs were collected. There were no refusals of nasal or oral swab collection.

Nasal samples were collected by moistening the swab in 0.9% saline (sterile technique) and introducing it into both nostrils until the maximum depth that could be tolerated by the participant. The rod was rotated, gently pressing the end against the mucosa. For oropharyngeal sampling, the swab was gently pressed and rotated over the tonsils and behind the uvula (posterior pharynx), avoiding touching the tongue, buccal mucosa, and uvula. For the collection of rectal samples, the tip of the swab was passed approximately 2 cm from the anal sphincter, rotating it carefully to collect a sample from the anal crypts and ensuring that there was fecal staining on the cotton after removal of the swab. After collection, the swabs were transported in Stuart’s medium to the Laboratory of Microbiology, Department of Microbiology and Immunology, Botucatu Institute of Biosciences.

### 4.4. Phenotypic Identification of S. aureus

The samples collected from the three sites were seeded onto Petri dishes containing Baird Parker agar, a selective medium for *Staphylococcus*. After incubation for 48 h at 37 °C, the colonies growing on the agar were submitted to Gram staining for assessing purity, observation of morphology, and specific staining. After confirmation of these features, the tube catalase and coagulase tests were performed according to Koneman et al. [34], as well as additional biochemical tests (maltose, trehalose, and mannitol fermentation).

### 4.5. Genotypic Identification of S. aureus

DNA was extracted using the Illustra Kit (GE Healthcare, Little Chalfont, Buckinghamshire, United Kingdom). After extraction, PCR was performed for amplification of the *Sa442* DNA fragments, which is specific for *S. aureus*, following the protocol described by Martineau et al. [35]. The following primers that amplify a 241-bp fragment were used: *Sa442*-1 (5′-AAT CTT TGT CGG TAC ACG ATA TTC TTC ACG-3′) and *Sa442*-2 (5′-CGT AAT GAG ATT TCA GTA GAT AAT ACA ACA-3′).

### 4.6. Antimicrobial Susceptibility Tests

All isolates were submitted to antimicrobial susceptibility testing by the disk diffusion method according to the criteria recommended by the Clinical Laboratory Standards Institute (CLSI) [36]. Cultures in BHI broth previously incubated for 18–24 h and adjusted with saline to a 0.5 McFarland turbidity standard were used for the preparation of the inoculum. The following drugs were tested: oxacillin (1 µg), cefoxitin (30 µg), linezolid (30 µg), quinupristin/dalfopristin (15 μg), and sulfamethoxazole/trimethoprim (25 µg). After density adjustment, the inoculum was seeded with a sterile swab on Mueller-Hinton agar, and the drug-impregnated disks were placed on the agar surface. The plates were incubated for 24 h at 35 °C. Antimicrobial activity was evaluated based on the diameter of the inhibition halo, which was interpreted according to the CLSI criteria. The *S. aureus* ATCC 25923 reference strain was used as a control during the test.

### 4.7. Determination of Minimum Inhibitory Concentration (MIC)

The vancomycin MIC was determined by the E-test. This quantitative method uses inert and transparent plastic strips (60 mm long and 5.5 mm wide) that carry a concentration gradient of the stabilized antimicrobial agent. The results were analyzed following the definitions established by the CLSI [36].

### 4.8. Molecular Detection of the mecA Gene and Characterization of SCCmec

The PCR assays for the detection of the *mecA* gene (methicillin resistance gene) were performed following the parameters described by Murakami et al. [37]. The following primers that amplify a 533-bp fragment were used: *mecA*1 (5′-AAA ATC GAT GGT AAA GGT TGG C-3′) and *mecA*2 (5′-AGT TCT GCA GTA CCG GAT TTG C-3′). International reference strains were included as positive (*S. aureus* ATCC 33591) and negative controls (*S. aureus* ATCC 25923) in all reactions. The efficiency of the amplifications was monitored by electrophoresis of the reaction on SYBR Safe-stained agarose gel (2%).

SCC*mec* typing was performed by multiplex PCR as described by Oliveira and de Lencastre [38] and updated by Milheiriço et al. [17]. The following reference strains were used: COL for SCC*mec* type I; N315 for SCC*mec* type IA; PER34 for SCC*mec* type II; AN546 for SCC*mec* type III; HU25 for SCC*mec* type IIIA, and MW2 for SCC*mec* type IV.

### 4.9. Pulsed-Field Gel Electrophoresis (PFGE)

All *mecA* gene-positive isolates obtained in this study were submitted to molecular typing by PFGE, following the modified protocol of McDougal et al. [39]. The MRSA isolates were cultured in BHI broth for 24 h. Next, 400 µL of the sample was added to a microtube and centrifuged at 12,000 rpm for 50 s. The supernatant was discarded, and 300 μL TE solution (10 mM Tris, 1 mM EDTA [pH 8.0]) was added. The samples were left in a water bath at 37 °C for 10 min. After vortexing, 5 μL lysostaphin (1 mg/mL in 20 mM sodium acetate [pH 4.5]) and 300 μL low-melt agarose were added. Agarose plugs of the samples were then prepared. After solidification, the plugs were transferred to a 24-well plate containing 2 mL EC solution (6 mM Tris-HCl, 1 M NaCl, 100 mM EDTA, 0.5% Brij-58, 0.2% sodium deoxycholate, 0.5% laurylsarcosine sodium) and incubated for at least 4 h at 37 °C. After this period, the EC solution was removed, and the plugs were washed four times (intervals of 30 min) with 2 mL TE at room temperature.

The *SmaI* enzyme (Fast Digest *SmaI*, MBI Fermentas Inc. Hamilton, Ontario, Canada) was used for genomic DNA restriction. Electrophoresis on 1% agarose gel prepared with 0.5 M TBE (Pulsed Field Certified Agarose, BioRad Laboratories, Hercules, CA, USA) was carried out in a CHEF-DR III System (BioRad Laboratories, Hercules, CA, USA) under the following conditions: pulse time of 5–40 s for 21 h; linear ramp; 6 V/cm; angle of 120°; 14 °C; 0.5 M TBE as running buffer. The Lambda Ladder PFG (New England BioLabs, Ipswich, Massachusetts, EUA) was used as a molecular marker. The gel was stained with GelRed (10,000× in water; Biotium, Fremont, CA, USA) for 1 h and photographed under UV transillumination.

The BioNumerics software (version 7.6; Applied Maths, Sint-Martens-Latem, Belgium) was used for similarity analysis. The dendrogram was constructed by the UPGMA method (Unweighted Pair Group Method with Arithmetic Mean), adopting a band position tolerance of 1.2% and optimization of 1%. A dice similarity coefficient ≥ 80% was chosen for the definition of clusters.

The *ApaI* restriction enzyme was used for MRSA isolates that could not be typed after digestion with *SmaI*.

### 4.10. Multilocus Sequence Typing (MLST)

MLST was performed as described by Enright et al. [40]. Each primer pair amplifies an internal fragment of the housekeeping genes (about 500 bp). The following genes were used: carbamate kinase (*arcC*), shikimate dehydrogenase (*aroE*), glycerol kinase (*glpF*), guanylate kinase (*gmk*), phosphate acetyltransferase (*pta*), triosephosphate isomerase (*tpi*), and acetyl coenzyme A acetyltransferase (*yqiL*). The products were purified using the Real Biotech Corp (RBC) HiYield™ Gel/PCR Fragments Extraction Kit. The reactions were carried out in an ABI3500 8-capillary sequencer (50 cm) using POP7 (Applied Biosystems, Waltham, Massachusetts, USA) as polymer. The sequences (electropherograms) were visualized using the BioNumerics program (version 7.6; Applied Maths, Sint-Martens-Latem, Belgium). The sequences were analyzed and compared via an online database (https://pubmlst.org) (accessed on 29 August 2020).

### 4.11. Statistical Analysis

The study design is cross-sectional, and the presence of *S. aureus* or MRSA was defined as the outcome. For the identification of risk factors, univariate analysis was first performed. Dichotomous variables were analyzed using the chi-squared test or Fisher’s exact test when appropriate. Numerical variables were compared by the Mann-Whitney U test. We then tested confounding in multivariate logistic regression models using a stepwise forward selection strategy, with *p* < 0.1 being adopted as a criterion for entry and continuation of the variables in the models. The data were stored in EPI INFO 7 (Centers for Disease Control and Prevention, Atlanta, GA, USA), and all analyses were performed using SPSS 20 (IBM, Armonk, NY, USA).

## Figures and Tables

**Table 1:** Prevalence of *S. aureus* and MRSA in long-term care facilities.

		LTCF																	
Prevalence		A (N = 7) Small		B (N = 6) Small		C (N = 12) Small		D (N = 6) Small		E (N = 7) Small		F (N = 18) Medium		G (N = 60) Large		H (N = 16) Medium		I (N = 18) Medium	
		N	%	N	%	N	%	N	%	N	%	N	%	N	%	N	%	N	%
Total	*S. aureus*	6	85.7	3	50.0	7	58.3	2	33.3	3	42.9	5	27.7	20	33.3	8	50.0	3	16.6
	MRSA	1	14.3	1	16.7	0	0	0	0	0	0	0	0	8	13.3	4	25.0	0	0
Nasal	*S. aureus*	4	57.1	1	16.7	1	8.3	2	33.3	0	0	2	11.1	12	20.0	3	18.7	2	11.1
	MRSA	0	0	0	0	0	0	0	0	0	0	0	0	4	6.6	2	12.5	0	0
Oral	*S. aureus*	1	14.3	1	16.7	3	25.0	0	0	1	14.3	0	0	3	5.0	3	18.7	1	5.5
	MRSA	1	14.3	1	16.7	0	0	0	0	0	0	0	0	3	5.0	2	12.5	0	0
Rectal	*S. aureus*	0	0	0	0	0	0	0	0	1	14.3	2	11.1	0	0	0	0	0	0
	MRSA	0	0	0	0	0	0	0	0	0	0	0	0	0	0	0	0	0	0
Nasal + Oral	*S. aureus*	1	14.3	1	16.7	2	16.7	0	0	0	0	1	5.5	3	5.0	2	12.5	0	0
	MRSA	0	0	0	0	0	0	0	0	0	0	0	0	1	1.6	0	0	0	0
Nasal + Rectal	*S. aureus*	0	0	0	0	1	8.3	0	0	0	0	0	0	2	3.3	0	0	0	0
	MRSA	0	0	0	0	0	0	0	0	0	0	0	0	0	0	0	0	0	0
Oral + Rectal	*S. aureus*	0	0	0	0	0	0	0	0	1	14.3	0	0	0	0	0	0	0	0
	MRSA	0	0	0	0	0	0	0	0	0	0	0	0	0	0	0	0	0	0

Note: Prevalence of *S. aureus* and methicillin-resistant *S. aureus* (MRSA) in nine long-term care facilities for the elderly (LTCF), designated A to I.

**Table 2:** Antimicrobial susceptibility profile of MSSA and MRSA isolated from bedridden or institutionalized individuals.

*S. aureus* (*n* = 97)	*mecA* Gene	Oxacillin		Cefoxitin		Linezolid		Q/D		S/T		Vancomycin MIC # (µg/mL)
		R	S	R	S	R	S	R	S	R	S	
MSSA (*n* = 77)	0	3 *	74	0	77	0	77	0	77	0	77	0.19–1.5
MRSA (*n* = 20)	20	13	7 **	13	7 **	0	20	0	20	0	20	0.19–1.5
Total (*n* = 97)	20	16	81	13	84	0	97	0	97	0	97	0.19–1.5

Note: Isolates identified as MSSA and MRSA based on the presence of the *mecA* gene. R: resistant. S: susceptible. Q/D: quinupristin/dalfopristin. S/T: sulfamethoxazole/trimethoprim. MIC: minimum inhibitory concentration. # Range of vancomycin susceptibility obtained for *mecA* gene-negative (MSSA) and -positive (MRSA) isolates. * Isolates resistant to oxacillin that did not carry the *mecA*. ** Isolates susceptible to oxacillin and cefoxitin that carried the *mecA* gene.

**Table 3:** Univariate and multivariate analysis (logistic regression model) of predictors of *S. aureus* carriage.

Risk Factors	Univariate Analysis				Multivariate Analysis	
	*S. aureus* (*n* = 76)	Negative (*n* = 150)	OR (95 %CI)	*p*	OR (95 %CI)	*p*
Demographic data						
Male gender	**37 (48.7)**	**48 (30.7)**	**2.15 (1.22–3.79)**	**0.008**	**2.59 (1.41–4.76)**	**0.002**
Age [years], median (quartile)	77.5 (70–84)	80 (70–85)	…	0.41		
Living in a long-term care facility	56 (73.7)	94 (62.7)	1.6 (0.908–3.065)	0.98	**2.05 (1.07–3.91)**	**0.03**
Time at risk * [months], median (quartile)	**36 (12–66)**	**66 (24–66)**	**…**	**0.003**		
Comorbidities						
Heart disease	3 (3.9)	17 (11.3)	0.32 (0.01–1.13)	0.07	**0.18 (0.05–0.70)**	**0.01**
Lung disease	5 (6.6)	8 (5.3)	1.25 (0.40–3.96)	0.77		
Kidney disease	4 (5.3)	3 (2.0)	2.72 (0.59–12.48)	0.22		
Liver disease	0 (0.0)	2 (1.3)	0.00 (…–…)	0.55		
Diabetes mellitus	19 (25.0)	29 (19.3)	1.39 (0.72–2.68)	0.32		
Central nervous system disease	20 (26.3)	33 (22.0)	1.26 (0.66–2.40)	0.46		
Cancer	6 (7.9)	6 (4.0)	2.057 (0.64–6.60)	0.21		
AIDS	0 (0.0)	1 (0.7)	0.00 (…–…)	1.00		
Pressure ulcer	6 (7.9)	9 (6.0)	1.34 (0.46–3.92)	0.58		
Charlson Comorbidity Index, median (quartile)	1 (1–1)	1 (0–1)	…	0.25	**1.35 (1.01–1.92)**	**0.047**
Procedures						
Hospitalization **	14 (18.4)	19 (12.7)	1.55 (0.73–3.30)	0.24		
Surgery **	6 (7.9)	3 (2.0)	4.20 (1.020–17.28)	0.064	**5.99 (1.26–28.92)**	**0.02**
Other invasive procedures **	4 (5.3)	7 (4.7)	1.13 (0.32–4.004)	1.00		
Antimicrobial use **	10 (13.2)	9 (6.0)	2.37 (0.92–6.11)	0.67		

Note: Data are reported as percentages, except when otherwise specified. Significant results are shown in bold. OR: odds ratio. CI: confidence interval. * Time spent in a long-term care facility or bedridden. ** In the last 6 months.

**Table 4:** Univariate and multivariate analysis (logistic regression model) of predictors of MRSA carriage.

Risk Factors	Univariate Analysis				Multivariate Analysis	
	MRSA (*n* = 18)	Negative (*n* = 208)	OR (95 %CI)	*p*	OR (95 %CI)	*p*
Demographic data						
Male gender	**11 (61.1)**	**72 (34.6)**	**2.96 (1.103–7.98)**	**0.03**	**3.29 (1.18–9.17)**	**0.02**
Age [years], median (quartile)	76 (69.5–83)	77 (60–85)	…	0.51		
Living in a long-term care facility	14 (77.8)	136 (65.4)	1.85 (0.58–5.83)	0.28		
Time at risk * [months], median (quartile)	36 (18–67.5)	60 (18–66)		0.61		
Comorbidities						
Heart disease	2 (11.1)	18 (8.7)	1.31 (0.28–6.20)	0.66		
Lung disease	2 (11.1)	11 (5.3)	2.39 (0.45–10.98)	0.27		
Kidney disease	1 (5.6)	6 (2.9)	1.98 (0.22–17.41)	0.44		
Liver disease	0 (0.0)	2 (1.0)	0.00 (…–…)	1.00		
Diabetes mellitus	1 (5.6)	47 (22.6)	0.20 (0.26–1.55)	0.13		
Central nervous system disease	5 (27.8)	48 (23.1)	1.28 (0.43–3.77)	0.77		
Cancer	1 (5.6)	11 (5.3)	1.053 (0.12–8.65)	1.00		
AIDS	0 (0.0)	1 (0.5)	0.00 (…–…)	1.00		
Pressure ulcer	3 (16.7)	12 (5.8)	3.26 (0.83–12.85)	0.10		
Charlson Comorbidity Index, median (quartile)	1 (1–1.5)	1 (0–1)	…	0.91		
Procedures						
Hospitalization **	4 (22.2)	29 (13.9)	1.76 (0.54–5.73)	0.30		
Surgery **	1 (5.6)	8 (3.8)	1.47 (0.17–12.46)	0.53		
Other invasive procedures **	1 (5.6)	10 (4.8)	1.16 (0.14–9.65)	1.00		
Antimicrobial use **	2 (11.1)	17 (8.2)	1.40 (0.29–6.62)	0.65		

Note: Data are reported as percentages, except when otherwise specified. Significant results are shown in bold. OR: odds ratio. CI: confidence interval. * Time spent in a long-term care facility or bedridden. ** In the last 6 months.

## Data Availability

The data presented in this study are contained within the article.

## Appendix A

Sample size formula

A representative sample was selected based on the following parameters:

- Population size (finite population correlation factor or FCP) (N): 350;
- Hypothetical % frequency of the outcome factor in the population (p): 33% ± 5;
- Confidence limits as % of 100 (absolute ±%) (d): 5%;
- Design effect (DEFF): 1;
- Equation: n = [DEFF ∗ Np(1 − p)]/[(d2/Z21 − α/2 ∗ (N − 1) + p ∗ (1 − p)];
- Sample size (95% confidence interval): 173.
